# Understanding interleukin 11 as a disease gene and therapeutic target

**DOI:** 10.1042/BCJ20220160

**Published:** 2023-12-06

**Authors:** Stuart A. Cook

**Affiliations:** 1 MRC-London Institute of Medical Sciences, Hammersmith Hospital Campus, London, U.K.; 2 National Heart Research Institute Singapore, National Heart Centre Singapore, Singapore, Singapore; 3 Cardiovascular and Metabolic Disorders Program, Duke-National University of Singapore Medical School, Singapore, Singapore

**Keywords:** fibrosis, inflammation, therapeutics

## Abstract

Interleukin 11 (IL11) is an elusive member of the IL6 family of cytokines. While initially thought to be a haematopoietic and cytoprotective factor, more recent data show instead that IL11 is redundant for haematopoiesis and toxic. In this review, the reasons that led to the original misunderstandings of IL11 biology, which are now understandable, are explained with particular attention on the use of recombinant human IL11 in mice and humans. Following tissue injury, as part of an evolutionary ancient homeostatic response, IL11 is secreted from damaged mammalian cells to signal via JAK/STAT3, ERK/P90RSK, LKB1/mTOR and GSK3β/SNAI1 in autocrine and paracrine. This activates a program of mesenchymal transition of epithelial, stromal, and endothelial cells to cause inflammation, fibrosis, and stalled endogenous tissue repair, leading to organ failure. The role of IL11 signalling in cell- and organ-specific pathobiology is described, the large unknowns about IL11 biology are discussed and the promise of targeting IL11 signalling as a therapeutic approach is reviewed.

## Introduction

The purpose of this review article is to outline the paradigm shift in our understanding of Interleukin 11 (IL11) biology that has taken place very recently [1–4]. *Paradigm shift* is an overused term but in truth we believe it applies to the IL11 field as, while IL11 was until recently accepted by the scientific community as anti-fibrotic, anti-inflammatory, and pro-regenerative; newer studies highlight that IL11 is in fact the opposite: pro-fibrotic, pro-inflammatory and anti-regenerative. How can this be so? To explain this conundrum, I highlight two major misunderstandings of IL11 biology that arose due to incorrect interpretations of earlier studies in mice and humans. I discuss the more recent understanding of the complexities of IL11 signalling across cell types and highlight insights into IL11 biology from human and mouse genetics, while exploring the evolutionary context for IL11 function. The role of IL11 in disease pathobiology in individual organs is critiqued, the [many] outstanding uncertainties about IL11 biology are mentioned and I end with a review of the current clinical trials of anti-IL11 therapeutics.

## Discovery of IL11 and a first major misunderstanding

IL11 was first identified in 1990 as a factor secreted from a bone marrow-derived stromal cell lineage that supported the growth of an IL6-dependent murine plasmacytoma [5]. During its initial characterisation, IL11 was found to synergise with IL3 in megakaryocyte colony formation, which resulted in it being given a role in platelet production [6,7]. This belief was further established in studies that showed injection of recombinant human IL11 (rhIL11) to mice, monkeys and humans increased platelet counts [8]. Subsequent clinical trials in patients with chemotherapy-induced thrombocytopenia confirmed the efficacy of rhIL11 for increasing platelet counts and rhIL11 (Neumega; (Oprelvekin)) was approved by the FDA in 1998 for treating thrombocytopenia [9–11].

I think it is accurate to say that since the late 1990s IL11 has been thought, by most, as particularly important for platelet biology. However, even as early as 1997 there were data to suggest that the primary function of IL11 was unrelated to haematopoiesis as deletion of the mouse Il11 receptor (*Il11ra1*) had no effect on blood cell parameters either at baseline or in response to bone marrow toxicity or haemolytic stresses [12]. Despite this evidence, Neumega use in patients expanded around the world and was additionally trialled in patients with low platelets due to cirrhosis [13], myelodysplastic syndrome [14], von Willebrand factor deficiency [15], dengue fever [16] and sepsis [17].

In more recent studies, in mice deleted for *Il11*, mice administered neutralising IL11 or IL11RA antibodies for long periods and in human IL11RA knockouts, there have been no reported effects of IL11 loss-of-function (LOF) on blood counts, in keeping with the early observations in the *Il11ra1* deleted mouse [12,18–21].

This is not to say that endogenous IL11 has no role in haematopoiesis but suggests that acute thrombocytosis following injection of high dose rhIL11 does not reflect its true biology. How rhIL11 induces platelet counts remains to be discerned but oncostatin M (OSM, another IL6 family cytokine) is important for haematopoiesis and perhaps high dose rhIL11 activates gp130-related JAK/STAT signalling in the bone marrow, similar to OSM [22,23]. Irrespective of this, it can be concluded that the first major physiological function assigned to IL11 — as a haematopoietic factor — was incorrect.

## An evolutionary perspective

Following its discovery, IL11 was designated an IL6 family cytokine, which comprise OSM, IL6 itself, ciliary neurotrophic factor (CNTF), leukaemia inhibitory factor (LIF), cardiotrophin 1 (CT-1), IL31 and IL27. These cytokines mostly act by binding to varied, and sometimes shared, alpha receptors and then complex with the common gp130 coreceptor to initiate intracellular signalling, canonically JAK/STAT. The class I cytokine receptor gp130 (IL6ST) is evolutionarily very old with homologues found in sea squirt (>700 m years ago), whereas IL6 family receptors and cytokines emerged later (>400 m years ago) in the fish [24]. As such, IL11 evolved to carry out its primary biological function(s) in ancient fish.

In healthy fish, Il11 can be detected in the gills and intestine but is little expressed elsewhere. However, following viral or bacterial infection, Il11 levels are elevated systemically [25–27]. Il11 is also strongly up-regulated following injection of fish with a synthetic pathogen-associated molecular pattern protein, in an alarmin-type response [26]. It might therefore be thought that Il11's function in the fish is primarily immune related, although its up-regulation is most apparent in epithelial and stromal cells, not immune cells.

More recent data point to a possible alternative primary function of Il11 in fish: for fin, tail and scale regeneration [28,29]. This activity seems unique to Il11 that is specifically up-regulated in the regenerative organ, the blastema, where other IL6 family members are not. As mentioned in an editorial [28], *nothing in biology makes sense except in the light of evolution* and the question arises as to what evolutionary pressure prioritised Il11 for regeneration [30]. This cannot be answered, however recovery from predatory fin/scale damage is strongly advantageous for fish viability and fecundity and this evolutionary force may have helped shape early Il11 biology.

The case for a specific role for Il11 in epimorphic appendage regeneration is made stronger by data from other regenerative species. In Xenopus tadpoles following tail amputation, regenerative blastema cells uniquely express *il11* [26]. Furthermore, non-cell autonomous *il11* activity is essential for Xenopus tail regeneration and the Xenopus homologue of IL11RA *(il11ra.L*) is equally required for tail regeneration [31,32]. Supportive data are found in studies of the axolotl where conspecific bite injuries are common and limb regeneration enables injured animals to reach sexual maturity [33]. Integrative analysis of RNA-seq of regenerative fish and axolotl blastemas revealed that *Il11* is the most up-regulated gene shared between species and JAK/STAT is the topmost shared pathway [34].

It therefore seems likely that the main function of Il11 in early evolution was in regeneration. The mechanisms underlying IL11 effects in regeneration are discussed in a recent editorial [28] and include fibroblast mesenchymal transition, extracellular matrix remodelling, inflammation, cell dedifferentiation and cell migration [1,34]. A secondary role for IL11 in stromal immunity and epithelial barrier function appears possible but more nuanced and is currently less well defined. Arguing against a dominant role for IL11 in other important homeostatic/physiological processes: mature tadpoles and adult fish, mice and humans lacking IL11 signalling appear mostly fit and healthy [2,28,32].

## Species specificity and further misunderstanding

The foundational misconception that IL11 is a haematopoietic factor led to the development of rhIL11 as a drug to treat thrombocytopenia. Because of this, rhIL11 became readily available from the mid 1990s and was used widely in experiments. The administration of this new human-derived reagent to mice in studies for some 20 years — until the mid 2010s — showed that rhIL11 was anti-fibrotic, anti-inflammatory, and pro-regenerative across murine models of disease. By way of example, in the mouse rhIL11 protected against liver damage and promoted liver regeneration [35–40], protected from lung damage [41,42], reduced kidney inflammation [43–46], protected against arthritis [47], reduced colitis [48–52] and limited cardiac fibrosis and dysfunction [53–55].

These replicated and robust data were interpreted — as would be expected — as a read out of IL11 gain-of-function (GOF) in murine models of disease. These data were so convincing that, with extrapolation, rhIL11 was postulated as a therapy for human disease where rhIL11 had worked in a murine model of said disease. This led to a number of clinical trials where rhIL11 was administered to patients with myocardial infarction [56], colitis [57,58], liver disease [59], hepatitis [59], rheumatoid arthritis [60] and other conditions [2].The outcomes of these trials were mixed and none progressed to pivotal phase 3 studies, suggesting lack of efficacy or toxicities prevented progression of rhIL1 as therapy for these conditions.

In 2016, we first identified IL11 as the major transcriptional target of TGFβ in primary cultures of human heart fibroblasts [1]. On review of the literature, we fully expected its up-regulation to represent a feedback loop to limit cardiac fibroblast activation [53–55]. However, after a series of experiments, we discovered this was not the case. Indeed, we concluded the opposite: that IL11 activates fibrogenic protein translation in human fibroblasts, to cause fibrosis. Furthermore, injection of species matched recombinant mouse Il11 (rmIL11) to the mouse or transgenic expression of rmIL11 in fibroblasts in the adult mouse caused heart and kidney fibrosis and cardiorenal failure [1].

At the time, we ourselves and the reviewers of our manuscripts and grants asked, ‘how can IL11 be toxic, pro-fibrotic and pro-inflammatory when 20 years of data teaches the opposite?’. In truth, we did not know the answer, but we observed that rhIL11 had little effect on mouse fibroblasts activation, whereas rmIL11 was strongly fibrogenic in mouse cells.

Over the following years, we realised that whenever we identified a maladaptive effect of endogenous or species matched recombinant IL11 there was often a publication using rhIL11 in a relevant mouse model showing the opposite. It occurred to us that perhaps rhIL11 was somehow blocking the toxicities of endogenous mouse Il11 in mouse models of disease. This turned out to be the case, which we demonstrated in studies of toxin-induced liver damage where rhIL11 dose-dependently inhibited endogenous mouse Il11-dependent hepatotoxicity, which had in fact been observed earlier by others [37,61,62].

It transpires that rhIL11 binds strongly to mouse Il11ra1 but incompletely activates the resulting (rhIL11:Il11ra1:gp130) signalling complex, thus acting as a partial/incomplete agonist that blocks endogenous mouse Il11 signalling. Bizarrely, rhIL11 can partially activate gp130 signalling in mouse cells lacking Il11ra1. Of note, opposing effects of rhIL11 and endogenous mouse Il11 had been reported previously in a mouse model of arthritis [63].

There is likely more to this story than the simple argument made here and rhIL11 effects on mouse Il11ra1 are undoubtedly more complex than we currently appreciate. Crystal structure studies will be needed to help further understand these phenomena. It may be, indeed is likely, that rhIL11 sometimes has specific, Il11ra1-mediated effects in the mouse. In addition, in some instances species-matched murine Il11 has been shown to protect mice from disease, and thus lack of species-specificity does not always explain effects [64]. Further studies are needed in this area.

**Figure 1.**
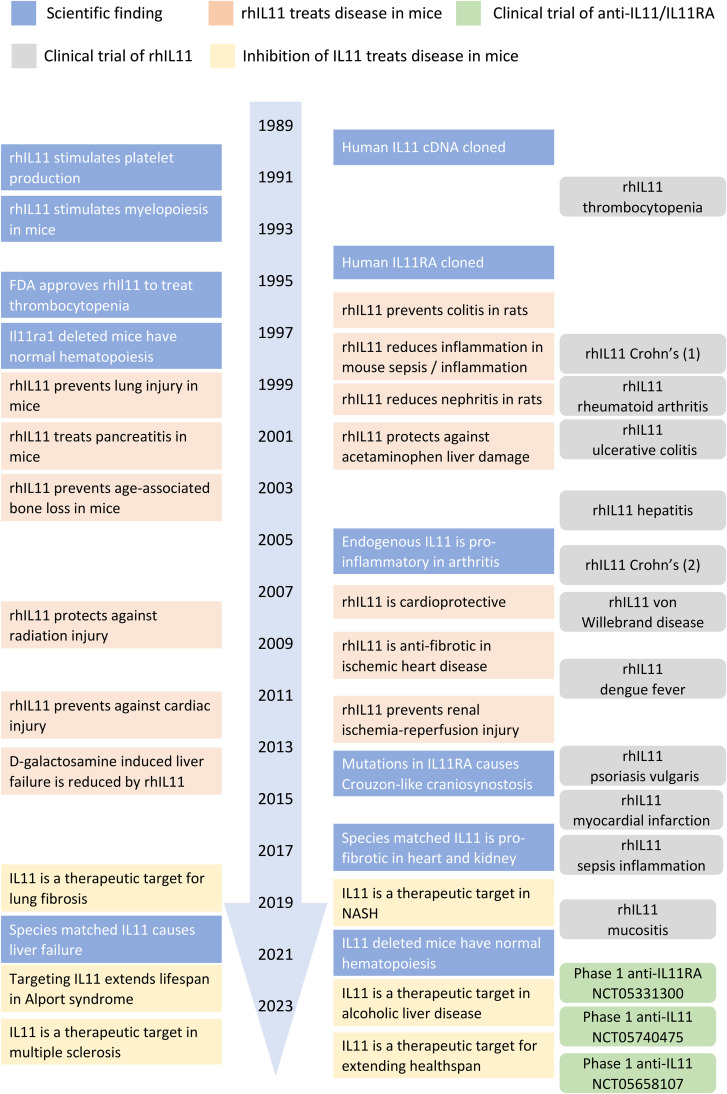
Overview of the evolution in our understanding of IL11 (patho)biology from its discovery in 1990 to present day. IL11 was initially thought important for haematopoiesis and a cytoprotective factor based mostly on experimental data generated using rhIL11 in mice. The conviction of its therapeutic properties led to a number of clinical trials in patients and it remains used to this day to treat thrombocytopenia. In 2017, IL11 was found to be toxic and subsequent proof-of-concept therapeutic studies across a range of mouse models of fibro-inflammatory diseases have led to clinical trials of anti-IL11 therapies.

To conclude, the early literature (1995–2015) correctly, exhaustively, and almost exclusively reported rhIL11 as a protective factor in mouse models of disease. However, the authors of these many studies did not appreciate that the effects that they observed largely reflected rhIL11-mediated inhibition of endogenous mouse Il11 function. As such, experiments using rhIL11 in mice, which were assumed a GOF, represented LOF experiments (Figure 1).

## IL11 signalling

To signal in *cis*, IL11 first binds to its cognate alpha receptor — IL11RA in humans, Il11ra1 in mice — and then binds to the shared gp130 (IL6ST) coreceptor, which dimerises with another IL11:IL11RA:gp130 molecule to form a hexameric signalling complex. This initiates canonical gp130-mediated signalling via JAK/STAT, notably JAK2/STAT3, which has been thought the primary IL11 pathway [65]. IL6 signals in a similar manner but it is notable that IL11RA and IL6R are expressed on different cell types with IL11RA most highly expressed on stromal cells (e.g. adipocytes, fibroblasts, vascular smooth muscle cells (VSMCs)) whereas IL6R is more strongly expressed on immune cells (e.g. monocytes) [1].

In cultured human fibroblasts, IL11 up-regulates a range of pro-fibrotic proteins in the absence of changes in their transcript abundance [1,66]. This intriguing phenomenon, which has yet to be fully described, suggested that IL11 signals via pathways other than just JAK/STAT and transcript abundance regulation. In our studies, and those of others, IL11-stimulated MEK/ERK activation has been identified as particularly important for fibroblast mesenchymal (myofibroblast) transition (FMT) and also VSMC mesenchymal transition (VMT), referred to as phenotypic switching [67–69]. MEK/ERK is a recognised non-canonical signalling pathway downstream of gp130 and is activated in a biphasic manner by IL11 *in vitro* with an initial phase that can be attenuated by increased DUSP activity and a later phase that is sustained [70–74]

As expected, IL11 activates JAK/STAT3 in human fibroblasts and epithelial cells (e.g. renal tubular epithelial cells (TECs) and hepatocytes) [70–72,74]. While IL11-stimulated ERK activation is biphasic and prolonged, the activation of JAK/STAT in cultured fibroblasts and TECs is immediate, transient and lesser in magnitude when compared with the effects of IL6 or OSM.

In fibroblasts, IL11-dependent JAK/STAT3 activity leads to the up-regulation of a range of pro-inflammatory factors such as *SERPINB2, TNFRSF18, IL33*, *CCL20, IL1RL1*, *CXCL3/5/8* and *ICAM1* [72]. While the earlier literature [43,47–49,75–77], and even recent reviews [78], suggested IL11 to be anti-inflammatory the newer data, *in vitro* and *in vivo*, strongly point to its pro-inflammatory effects [61,66,72]. Akt has been implicated in IL11 signalling but is inconsistently activated in IL11-stimulated fibroblasts [72]. We, and others, have documented JNK activation following IL11 stimulation of some cell types (e.g. hepatocytes) but this likely represents a secondary/indirect event [61]. In TECs, IL11 stimulates rapid, STAT3-mediated up-regulation of *IL33*, *CCL20* and *IL8* and down-regulation of TEC-specific genes, such as renal tubular urea transporter (SLC14A2) and aquaporin 6 (AQP6) [70].

Activation of MEK/ERK is a known pro-fibrotic pathway but it was not clear to us how IL11-stimulated ERK activation led to fibrogenic protein translation. Inhibition of AMPK and activation of mTOR plays an important role in fibrosis [79,80] and, in previous studies, ERK/P90RSK was shown to inhibit LKB1/AMPK [81]. We went on to show that IL11 stimulates ERK and P90RSK to dually phosphorylate LKB1, at serine 325 and 428, respectively, to inactivate LKB1, inhibiting AMPK and activating mTORC1 [73]. Identification of this IL11/ERK/LKB1/AMPK/mTOR pathway brings into focus a key pro-fibrotic signalling module that involves established and important pro-fibrotic signalling factors. It is important to mention here that the functional importance of phosphorylation of LKB1 at serine 325 and 428 in this pathway has yet to be established and requires new genetic models.

IL11-induced ERK/P90RSK activity has a second and important axis of influence — through the dual phosphorylation and inactivation of an alternative substrate: GSK3β. In this instance, ERK and p90RSK, dually phosphorylate GSK3β (Thr43 and Ser9, respectively) causing its inactivation [82]. This has the consequent effect of releasing SNAI1 from GSK3β-mediated repression and results in SNAI1-mediated down-regulation of the canonical epithelial marker, E-cadherin [83]. Thus, the IL11/ERK/P90RSK axis, by inactivating GSK3β, controls the SNAI1:E-cadherin switch, which is important for epithelial mesenchymal transition (EMT) and, in a related fashion, FMT of fibroblasts.

*Trans*-signalling is a field of IL6 biology and proposes that IL6 when complexed with soluble IL6R signals via gp130. The existence of IL6 *trans*-signalling relies in part on the use of a fusion protein termed HyperIL6, which has variable pathogenic or cytoprotective properties [62,84]. It also depends on the use of a decoy protein made from a soluble portion of gp130 linked to an antibody Fc domain (sgp130Fc) [85]. While sgp130Fc is taught as specific for IL6 *trans*-signalling, it inhibits CNTF *trans*-signalling as well as OSM and LIF *cis*-signalling and could, in theory, inhibit IL6 and IL11 *cis*-signalling [86,87]. Given these vagaries, and the conflicting reports of the existence or absence of IL11 *trans*-signalling [21,84,88], we take the view that the current data support IL11 *cis*-signalling as the dominant form of IL11 activity and believe that more work is needed on *trans*-signalling.

**Figure 2.**
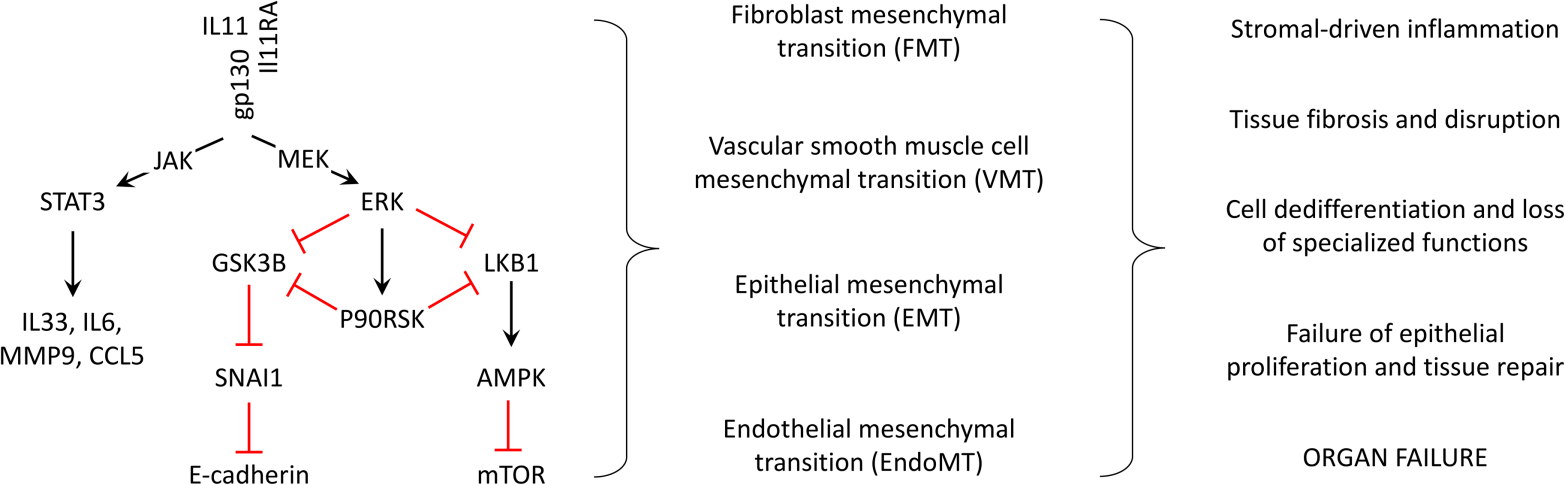
IL11-activated signalling, cellular mesenchymal transitions and organ-level pathologies. IL11 is not detected in health but is up-regulated, as an alarmin-like response to toxic, genetic, infective, mechanical, oxidative or metabolic disease factors. IL11 signalling pathways converge to cause mesenchymal transitions and a pro-inflammatory state across cell types. This leads to stromal inflammation, fibrosis, loss of specialised cellular functions, failed endogenous repair mechanisms and organ failure. Red connectors: inhibitory effects.

To summarise, IL11 signals in a range of stromal and epithelial cells, in both autocrine and paracrine, to (1) activate JAK/STAT3 that leads to increase the expression of pro-inflammatory genes, which is short-lived *in vitro,* and (2) more persistently activate ERK/P90RSK that together inhibit LKB1, to activate mTOR, and also inhibit GSK3β, to activate the SNAI1:E-Cadherin switch. Overall, these signalling events drive a program of mesenchymal gene expression and inflammation across cell types (Figure 2).

## Cellular pathobiology of IL11

### Stromal cells

The first cellular pathology linked with IL11 was its pro-fibrotic effect in fibroblasts, which it drives towards a myofibroblast state (via FMT) with the consequent secretion of extracellular matrix and pro-inflammatory factors (e.g. IL33, IL6, IL8, CCL20, CXCL3/5/8) [2]. This autocrine and self-amplifying process is accompanied by increased fibroblast migration and invasion: IL11 is specifically associated with invasive fibroblasts in idiopathic pulmonary fibrosis (IPF) [66,89]. In lung diseases, IL11 is associated with fibroblast senescence that is thought important for fibrosis [68,90,91].

VSMCs are another stromal cell type that highly express IL11RA at baseline and respond to IL11 in autocrine and paracrine [67,92]. IL11 causes VSMC dedifferentiation into a myofibroblast-like state (via VMT) with increased expression of mesenchymal and pro-inflammatory genes and loss of specialised contractile genes. *In vivo*, secretion of IL11 from VSMCs is associated with fibro-inflammation across tissues [92]. IL11 also induces VSMC senescence that is associated with pulmonary hypertension and fibrosis [90].

Pancreatic and hepatic stellate cells have fibroblast-like functions and are major determinants of fibrosis in the pancreas and liver. These cells express IL11RA at baseline and both secrete and respond to IL11 when stimulated with a range of disease factors, including mechanical stress [21,93–96]. IL11 signals in stellate cells to activate STAT3 and ERK that induces mesenchymal transitions leading to fibro-inflammatory diseases of the liver and pancreas [93,94].

### Polarised cells

Renal TECs proliferate in the response to kidney damage, which is centrally important for renal repair/regeneration [97]. When kidney damage is severe/repeated, TECs undergo SNAI1-driven partial EMT (pEMT) that prevents homeostatic replication and leads to renal failure [98,99]. In TECs, IL11 activates ERK/P90RSK to dually phosphorylate and inactive GSK3β [83,100] to cause SNAI1 up-regulation with resultant loss of specialised functions and proliferative capacity [70,74,101]. *In vitro*, IL11 strongly induces a STAT3-dependent pro-inflammatory transcriptional response (including: IL33, IL8 and CCL20) in TECs [70].

Following lung injury, regeneration of damaged alveolar epithelial cells occurs when alveolar epithelial type 2 (AT2) cells, and perhaps other progenitors, transform into alveolar epithelial type 1 (AT1) cells, which occurs via an intermediate KRT8+ cell state [102]. In the diseased lung, precision cut lung slices, organoids and cultured AT2 cells, IL11 disrupts alveolar epithelial cell regeneration by stalling reparative AT2 cells in the mesenchymal KRT8+ state [103–105]. Similarly, IL-11 transforms tracheal epithelial cells to a mesenchymal state which is associated with GSK3β phosphorylation cellular dysfunction [106].

Hepatocytes express IL11RA at baseline and secrete large amounts of IL11 in response to toxic injury, reactive oxygen species or fat loading [21,35,61,62,95,107,108]. Exposure of hepatocytes to IL11 causes cell death associated with activation of ERK, JNK and NOX4. In a mouse model of acetaminophen (APAP)-induced hepatotoxicity, hepatocyte-specific deletion of *Il11ra1*, *Il11* or *gp130* promotes hepatocyte regeneration and liver repair [61,62,109]. Of note, hepatocyte regeneration is inhibited by SNAI1 expression [70,110].

IL11 is secreted by endothelial cells (ECs) following their exposure to toxins or viral infection [111,112]. ECs respond to IL11, in autocrine or paracrine, by undergoing endothelial mesenchymal transition (EndoMT), which appears both STAT and ERK regulated [90]. In the context of rheumatoid arthritis and diabetic eye disease, IL11 secreted from fibroblasts causes EndoMT and this is linked with neo-angiogenesis [113,114].

### Cardiomyocytes

Cardiomyocytes are terminally differentiated heart muscle cells. These cells highly express IL11RA. While a number of studies show that species unmatched rhIL11 is protective against apoptosis in rat cardiomyocytes and beneficial in the heart generally [53–55,115], data from our group, and others, challenge these findings [1,69,116–118]. In a recent study, we show that injection of rmIL11 to mice causes dose-dependent impairment of heart contraction, which is due to a direct Il11ra1-mediated effect of rmIL11 in cardiomyocytes [119]. Thus, IL11 is toxic to cardiomyocytes.

### Bone cells

The situation regarding IL11 signalling and osteoblast, osteocyte, osteoclast activity and bone homeostasis is complex and difficult to resolve. These complexities are apparent in *IL11RA* mutant mice and humans that have variable penetrance and expressivity of developmental abnormalities of skull sutures, jaw, and tooth eruption, as well as shorter stature. IL11 deleted mice do not appear to share these bony abnormalities and mice with a particular gp130 mutation that selectively inhibits IL11 signalling have normal bones. IL11 has variably been thought to stimulate or inhibit bone formation in the adult and there is redundancy of gp130-binding cytokines in bone metabolism [120–123]. These matters remain to be dissected more fully.

### Immune cells

As compared with stromal or epithelial cells, immune cells express little IL11RA and IL11 signalling in monocytes seems redundant for some infectious processes [124]. IL11 function in immune cells may be pertinent for inflammation in autoimmune diseases and it was recently found important for NLRP3 activation in monocytes and inflammatory cell migration [125]. In the periphery, IL11RA and IL11 are most highly expressed in myeloid cells, notably neutrophils, although it may not directly stimulate neutrophil activity [126]. CD4+ T-cells from patients with multiple sclerosis also express IL11RA [127]. IL11 produced by tumour stromal cells suppresses the secretion of pro-inflammatory cytokines (e.g. IFNγ, TNFα, IL6) from CD4+ T-cells and reduces CD+ T cell infiltration, thus promoting immune evasion [128,129].

## A confluence of physiology and pathobiology across evolution?

It will be apparent to the reader that IL11-induced cellular pathobiologies in mammals — FMT, VMT, EMT and EndoMT — have notable similarities to its effects on cells in the blastema, in regenerative species [28]. As mentioned, the blastema is critically dependent on IL11 and comprises dedifferentiated and migrating epithelial/parenchymal/neuronal cells, which are in a mesenchymal-like state, and activated stromal cells, which are pro-inflammatory and secrete extracellular matrix [30,130]. Thus it appears that this ancient, IL11-driven mesenchymal program is activated in mammals, in response to injury. However, mammals do not generate blastemas and suffer chronic diseases of internal organs (endomorphic pathology) rather than epimorphic limb loss. Unfortunately, in mammalian species IL11 causes organ fibrosis, inflammation and failure. The primary function of IL11 in evolution, for epimorphic limb/fin regeneration, may explain its apparent redundancy for adult human health, as this function is not required for human fecundity.

## Human and mouse genetics of IL11 and IL11RA

Human gene knockouts are most informative of gene function and individuals with compound heterozygous LOF mutations of *IL11RA* are well described. These people can have craniosynostosis, conductive hearing loss, maxillary hypoplasia, exophthalmos, delayed tooth eruption, scoliosis and joint laxity, although some of these features may represent ascertainment bias in syndromic cases [18–20,131]. Humans with homozygous LOF in *IL11* itself have yet to be described but a human mutation in gp130 that causes selective impairment of IL11 signalling has been characterised: this causes variably penetrant craniosynostosis and retained deciduous teeth [132].

In the general population *IL11* and *IL11RA* have passively accumulated LOF mutations in the absence of selective constraint (see: https://gnomad.broadinstitute.org/), which suggests these genes are of little importance for human reproductive health. However, *IL11* and *IL11RA* have not been deleted/lost from the human genome and, even though a selective signal is not apparent in current databases, it seems some [not overly strong] aspect of IL11 biology is needed in humans, perhaps during development.

In contrast with *IL11* and *IL11RA*, LOF mutations in other IL6 family members and their cognate receptors are strongly selected against. Furthermore, human knockouts for IL6R have recurrent infections, eosinophilia, elevated IgE and eczema that highlights the very different biology of IL6 and IL11. Fittingly, humans with bi-allelic LOF mutation in gp130 that affects both IL11 and IL6 signalling present with combined immunodeficiency (IL6/OSM effect) and craniosynostosis (IL11 effect) [133].

In genome-wide and phenome-wide association studies, loci in proximity to *IL11* and *Il11RA*, and sometimes non-synonymous SNPs, have been recurrently linked with a few specific traits (see: https://genetics.opentargets.org/). For *IL11*, traits include reproductive span/menopause, height and osteoarthritis, although the directionality of effect of the SNPs remains to be resolved [134]. For *IL11RA*, traits include rheumatoid arthritis and height [135].

Mice with global deletion of *Il11ra1* were first described in 1997, when it was shown that IL11 signalling was redundant for haematopoiesis [12]. Female mice deleted for *Il11ra1* were found to be infertile due to a failure of embryo implantation and these mice can also have facial bone abnormalities and delayed tooth eruption [136,137]. The health of adult mice deleted for *Il11ra1* appears normal.

It is worth mentioning here that some of our misunderstanding of IL11 biology came about from studies in mice with global germline deletion of *Il11ra1* [12]. While this strain has been informative, there are differences between it and mice with global deletion of *Il11* or conditional and tissue-restricted *Il11ra1* deletion [138–140]. Perhaps the best example of this occurrence comes from liver studies: mice deleted for *Il11ra1* were shown not to be protected from toxin-induced liver injury [35], which we replicated [61]. However, mice with hepatocyte-specific deletion of *Il11ra1* [61], *Il11* [62] or gp130 [109] are protected from this form of liver injury, as are mice administered either anti-IL11, anti-IL11R or siRNA against *Il11* or *Il11ra1* [61,95,96]. It is therefore likely that global germline deletion of *Il11ra1* has secondary — direct or indirect — effects and insights derived using this strain should be validated with other approaches.

We, and others, generated mice with germline deletion of *Il11* and these mice do not have tooth, bone or snout issues but the females were found, as for *Il11ra1* deleted mice, to be infertile [138,139]. Haematological parameters are normal in *Il11* knockout mice, reinforcing the redundancy of IL11 for bone marrow homeostasis. Adult *Il11* knockout mice appear healthy. Mice with selective loss of IL11 signalling due to R279Q mutation of gp130 have normal cranial sutures, normal blood and bone parameters but incompletely penetrant snout abnormalities [132].

Overall, data from humans and mice with genetic LOF of IL11 signalling show a consistent pattern of mild and incompletely penetrant developmental abnormalities of the skull and facial bones along with delayed tooth eruption and variable skeletal abnormalities (e.g. reduced height, scoliosis and joint laxity). Differences in phenotypes associated with mutation in *IL11RA* or *IL11* are often suggested to be due to ‘*unknown extra ligands*’ that bind to IL11RA. While this is possible, it is equally likely, and supported by experimental findings in related contexts, that loss of IL11RA increases gp130 availability for other gp130 binding ligands/receptors that results in additional phenotypes [62,141,142].

## Heart disease

Based on studies using rhIL11, IL11 was initially thought cardioprotective, via a STAT3 mechanism [55], and subsequent experiments found rhIL11 to protect the mouse heart and limit fibrosis after a variety of injuries such that include ischaemia-reperfusion [53,55], infarction [54], and ischaemia [143]. These findings stimulated a small clinical trial of rhIL11 in patients with myocardial infarction [56].

However, in 2017 we discovered that IL11 causes cardiac fibrosis and dysfunction [1]. In follow-on studies, pharmacologic inhibition of IL11 signalling with anti-IL11 or anti-IL11RA antibodies reduced ERK-dependent cardiac fibrosis in pressure overload models [116,117]. GSK3α-mediated activation of ERK, an important driver of cardiac fibrosis, was found to operate independent of canonical TGFβ-SMAD3 signalling and, instead, be IL11-dependent [69]. Expression of species matched IL11 in the cardiomyocyte compartment leads to severe cardiac inflammation and fibrosis, cardiac EndoMT and ventricular dysfunction [118]. In patients, increased circulating levels of IL11 are associated with chronic heart failure [144]. IL11 levels are also elevated in patients with atrial fibrillation: IL11 causes atrial fibrosis in mice [145] and sensitises the atrium to fibrillation in old rats [146].

Acute atrial arrhythmia is the commonest side effect associated when rhIL11 is given to patients and serum BNP levels can reach levels diagnostic of heart failure in patients receiving rhIL11. Until recently, these phenomena were thought secondary to volume loading, which occurs with rnIL11 therapy [9,147]. However, we studied effects of rmIL11 in the mouse and have shown it to have direct toxicities in cardiomyocytes through on-target binding to Il11ra1. Injection of rmIL11 to the mouse causes dose-dependent cardiac impairment, increased inflammation (TNFα, NFκB and JAK/STAT) and disturbed calcium handling, with specific effects in the cardiomyocyte compartment [119]. As such, IL11 appears to be a direct cardiotoxin.

## Liver disease

**Figure 3.**
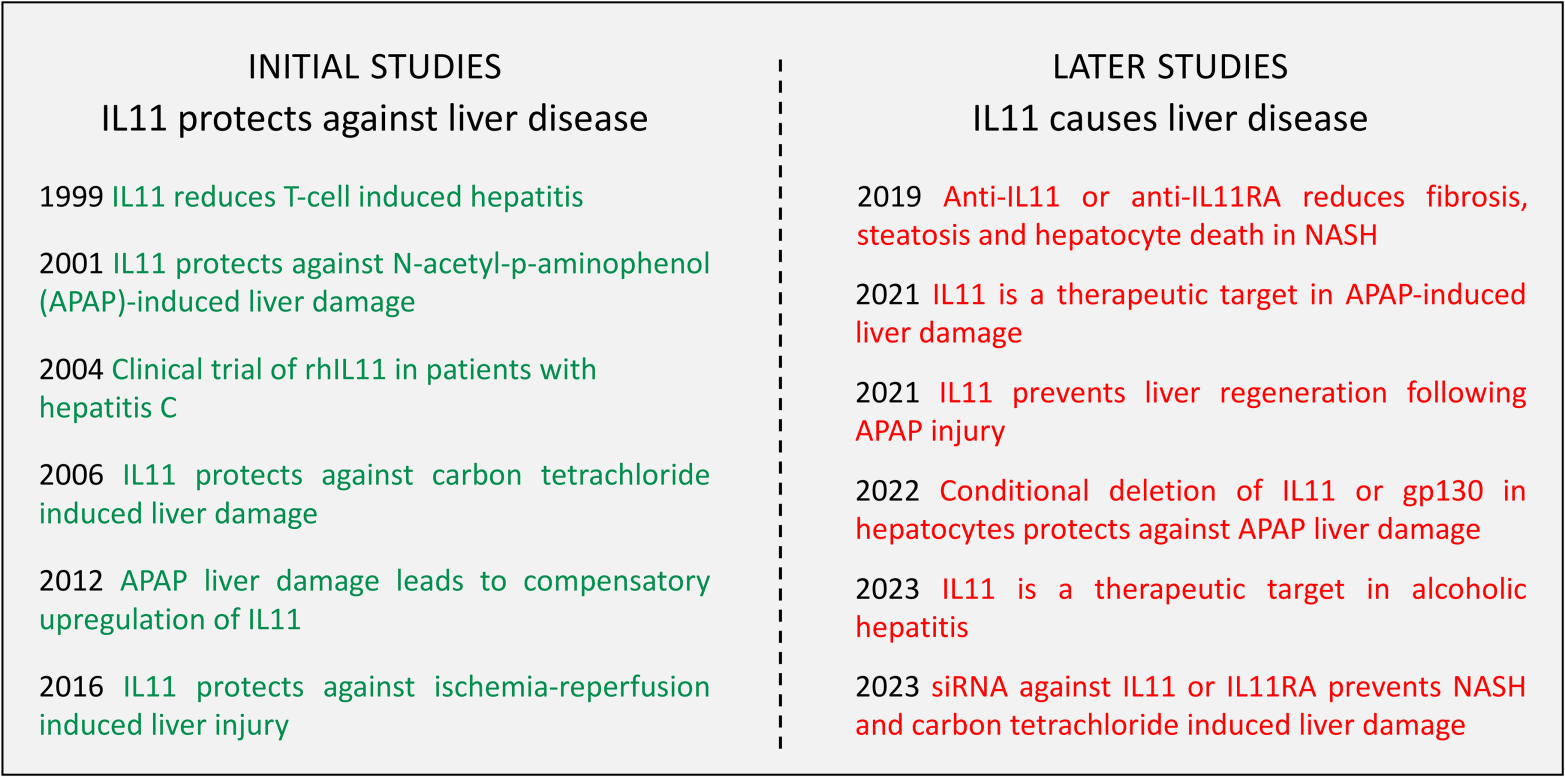
1999–2023, how our understanding of the role of IL11 in liver disease changed. Initial studies taught that IL11 is protective against immune-related hepatitis [40], APAP-induced liver failure [35,37], CCl_4_-induced liver damage [148] and ischaemia-reperfusion injury [39] that catalysed a clinical trial of rhIL11 as a treatment for patients with hepatitis C [59]. Later studies found instead that endogenous IL11 is hepatotoxic and that anti-IL11 therapy can be used to treat NASH and APAP-induced liver failure [21,61], IL11:IL11RA:gp130 signalling prevents liver regeneration [62,109], IL11 is a therapeutic target in alcoholic liver disease [107] and that siRNA against *Il11* or *Il11ra1* treats NASH and CCl_4_ liver toxicities [95,96].

Perhaps, of all organs, studies of the liver are most illustrative of the dichotomy between the early reports of IL11 effects in disease and the conclusions of more recent studies. This evolution in thinking is shown in Figure 3, which highlights some of the key publications on IL11 in the liver from the late 1990s through to 2023.

Of the experiments showing a beneficial effect of IL11 in liver disease, the 2001 study by Trepicchio that showed a protective effect of rhIl11 in APAP-induced murine liver failure caught our attention, as effects were acute, dose-dependent and we replicated the findings [37,61]. However, as we hypothesised, while rhIL11 was protective against toxin-induced liver damage in the mouse, rmIL11 was not. Strikingly, *in vitro* studies showed that rhIL11 binds to the mouse Il11ra1 receptor but does not faithfully activate it and thus protects mouse hepatocytes from rmIL11-induced cell death. This discovery changed our interpretation and understanding of earlier studies that used rhIL11 in mouse models.

IL11 pathobiology in the liver is complex and mediated through its activity in hepatocytes and hepatic stellate cells (HSCs). Initial hepatocyte damage, due to toxins, lipid loading or infective agents, causes IL11 secretion from hepatocytes that acts in autocrine to perpetuate cell damage through NOX4/JNK/caspase activation and also in paracrine, to stimulate HSC mesenchymal transitions and fibrosis [61,108]. These dual pathologies — damaged hepatocytes and activated HSCs — are seen in NASH and alcoholic liver disease and therapeutic targeting of IL11 signalling reduces disease in both these conditions [21,95,107].

Hepatocyte replication and liver regeneration is inhibited by IL11 and this pathology, related to SNAI1 up-regulation and hepatocyte partial EMT, is of importance for IL11-driven liver disease [110]. Gp130 signalling is more generally associated with failure of tissue regeneration, which further establishes the anti-regenerative effects of IL11:gp130 axis [149].

## Lung disease

In the lung, early studies showed up-regulation of IL11 following viral or bacterial infection, and in asthma [4]. However at that time, as in other organ systems, it was mostly suggested that the increased IL11 levels were compensatory, as transgenic expression of rhIL11 in the lung reduced lung inflammation and mortality in a mouse model of lung injury [41]. This same model exhibited lung inflammation/fibrosis [150] but transgenic expression of species-unmatched proteins can cause non-specific pathology and this observation was not followed up on [151]. In another study in rats, rhIL11 reduced LPS-induced lung TNFα levels, neutrophil infiltration, vasomotor dysfunction, and improved overall mortality [42]. After these publications in the 1990s, there were no major studies of IL11 in the lung for two decades.

In 2019, two contemporaneous manuscripts appeared on BioRxiv describing a role for IL11 in lung fibrosis [66,152]. The manuscript from our laboratory on IPF followed on from understandings of IL11 in our cardiac studies, whereas the identification of IL11 as a driver of lung fibrosis in Hermansky Pudlak syndrome (HPS) came about from unbiased RNA-seq screening. Together these studies identified IL11 secretion from stromal cells as an important for fibrosis in patients with IPF, in lung organoids from patients with HPS and in mice with bleomycin-induced lung fibrosis. In the HPS study, there was evidence of fibroblast-epithelial cross talk and of IL11-induced EMT. Importantly, we showed that anti-IL11 could prevent and reverse lung pathology.

The scientific community working on lung fibrosis was quick to embrace the idea that IL11 is an important pro-fibrotic factor and a number of publications have validated and extended findings: 2020, IL11/ERK signalling is important for senescence of lung cells [68] and fibroblast-specific expression of IL11 causes lung fibro-inflammation [140]; 2021, IL11 expression is associated with lung fibrosis in patients with rheumatoid arthritis [153], replication of IL11 up-regulation and its effects in bleomycin injury and HPS [154]; 2022, nanoparticle delivery of siRNA against *Il11* reduces lung fibrosis in the bleomycin mouse model [155], IL11 contributes to pulmonary artery remodelling and fibrosis in pulmonary hypertension [90], therapeutic targeting of IL11 reduces lung fibro-inflammation due to silica particle inhalation [156]; 2023, anti-IL11 reduces emphysematous disease in a mouse model of Marfan syndrome [157], IL11 disrupts alveolar epithelial cell repair functions by causing EMT [103,104], IL11 is elevated in interstitial lung disease in systemic sclerosis [158,159] and IL11 contributes to lung fibrosis in patients with SARS-COV-2 [160,161].

Given the large replication of the findings that IL11 underlies both common and rare forms of lung fibrosis through combined effects on lung epithelial and stromal cells and replicated therapeutic effects of anti-IL11 interventions, it is not surprising that the first clinical trials of anti-Il11 therapies are focused mainly on lung fibrosis, as discussed below.

## Kidney disease

IL11 levels are elevated in the kidney in a number of species in response to varied injuries that include diabetes [162,163], hypertension [164], ischaemia [165], toxins [1], infective agents (bacterial and viral) [27], preeclampsia [166,167] and obstructive nephropathy [70,165]. In addition, urinary IL11 levels are increased in patients with nephritis and IL11 is highly up-regulated in the kidneys of patients with end stage renal failure [168,169].

For the most part, IL11 up-regulation in the instances described above was thought protective based on functional studies using rhIL11 in mouse models of kidney disease [3,44,45]. In our study of 2017 [1], we observed the opposite: that injection of rmIL11 to mice caused renal fibrosis, that transgenic expression of rmIL11 in fibroblasts caused renal fibrosis and that mice deleted for *Il11ra1* are protected from renal fibrosis and impairment following toxin-induced kidney injury.

The potential of IL11 as a therapeutic target in kidney disease was subsequently demonstrated in two publications in 2022 [70,101]. These studies, showed (1) that IL11 is up-regulated in the kidneys of a mouse model of Alport syndrome and that this is associated with IL11-dependent, SNAI1-related pEMT of TECs, renal fibrosis and failure that can be treated with anti-IL11 therapy [101]. And (2), that IL11 up-regulation in a mouse model of CKD causes renal failure and that anti-IL11 therapy can reactivate endogenous renal repair mechanisms by reversing SNAI1-driven pEMTof TECs to promote kidney regeneration and recovery of function [70]. The findings of these two studies, with regard to IL11-induced EMT of TECs, were replicated and extended in a publication, which further validated IL11 as a therapeutic target using shRNA in an obstructive nephropathy model [74,170]. A recent study implicates IL11 release from macrophages in renal interstitial inflammation [171].

## Vascular disease

Our understanding of the role of IL11 in the vasculature is in its infancy but is gaining attention [172]. It has been shown that TGFβ1 and IL1α induce IL11 secretion from human aortic VSMCs and that IL11 inhibits VSMC proliferation following FGF stimulation [173,174]. Unbiased genome wide RNA studies showed that *IL11* is strongly increased in human coronary artery VSMCs following TGFβ1 stimulation [175].

In our own study of 2020, we observed large up-regulation of *IL11* in TGFβ-stimulated human aortic VSMCs and we showed expression of IL11RA in these cells [67]. Of note, IL11-induced VMT and VSMC phenotypic switching — from a differentiated contractile state to a mesenchymal secretory cell phenotype — and that IL11 was up-regulated in two models of arterial pressure overload and aortic remodelling, where anti-IL11 therapy reduced aortic inflammation, fibrosis and dilatation [67].

Remodelling of the vascular adventitia in response to angiotensin II is linked with IL11 activity and IL11 knockout mice are protected from disease-related vascular hypertrophy, adventitial fibrosis, and inflammation [176]. IL11 causes VSMC proliferation and vascular fibrosis of the pulmonary vasculature in the context of pulmonary hypertension [90]. IL11 is increased in the arterial wall in response to endothelial damage and anti-IL11 reduces vessel hyperplasia following arterial injury in the mouse [177]. In a mouse model of Marfan syndrome, which is characterised by elevated TGFβ activity due to LOF mutations in fibrillin-1, IL11 levels are increased and anti-IL11 therapy reduces aortic inflammation, matrix remodelling and dilatation [178]. In preeclampsia, IL11 levels are elevated and cause hypertension and spiral artery remodelling [166,167].

## Autoimmune diseases

### Multiple sclerosis

As with other conditions, early studies pointed to IL11 being beneficial in MS and for neuron myelination based on studies in *Il11ra1* deleted mice and the use of recombinant IL11 [179,180]. Later studies found that IL11 is increased in the blood and cerebrospinal fluid of patients with early stage MS symptoms and also in patients with relapsing-remitting MS, where blood levels, likely from IL11-secreting CD4+ cells, increased further during periods of clinical deterioration [127]. Furthermore, administration of IL11 to mice exacerbated clinical disease scores, inflammatory foci and brain-infiltrating CD4^+^ cells [181]. In single-cell RNA sequencing studies, IL11 had notable effects on monocytes where it up-regulated IL1β, NкKB and NLRP3. Therapeutic targeting of IL11 in mice with autoimmune encephalomyelitis lowered clinical scores and reduced brain infiltrates and demyelination [125].

### Systemic sclerosis

There is growing evidence for a pathogenic role for IL11 in skin and lung fibro-inflammation in systemic sclerosis (SSc). IL11 was first appreciated in SSc as the most up-regulated gene in pulmonary fibroblasts from patients with SSc interstitial lung disease (SSc-ILD) [182]. IL11 is also one the the most highly up-regulated genes in both resident and migratory skin fibroblasts from patients with SSc [183–185]. IL11 serum levels are elevated in SSc and are most increased in patients with SSc-ILD [158,159,186]. Of note, IL6 is also increased in SSc fibroblasts and anti-IL6R therapy was recently approved for the treatment of SSc-ILD, although this approach does not improve SSc skin disease [187]. IL33 is also implicated in SSc pathologies, and IL33 is strongly up-regulated by IL11 in fibroblasts [72,188].

### Rheumatoid arthritis

In rheumatoid arthritis (RA), IL-11 is elevated in synovial membranes, synovial fluid, and the blood, but was initially thought to be cytoprotective and anti-inflammatory based on the effects of rhIL11 in both human cells and in a mouse model of arthritis [47,189]. In an unique early study, endogenous IL11 was shown to be disease-causing in a mouse model of arthritis [63]. In gene expression studies, rheumatoid fibroblast-like synoviocyte cell lines stimulated with FasL strongly up-regulate (25-fold) *IL11* [190]. In synovial tissues from patients with RA, IL11 and IL11RA are up-regulated in synovial fibroblasts and IL11 secreted from fibroblasts stimulates ECs to promote neo-vascularisation [113]. Of note, IL11 levels, while increased in patients with RA in general, are specifically elevated in patients with active disease or RA-ILD [153].

### Inflammatory bowel disease

In early studies, the administration of rhIL11 was repeatedly shown to be protective against colon injury in mice and rats, across disease stimuli [48–52]. As recently as 2023, IL11 is proposed as protective against inflammatory bowel disease [52]. On the other hand, in patients with ulcerative colitis or Crohn's disease, IL11 is highly up-regulated in the colonic mucosa and its expression predicts lack of response to anti-TNFα therapy [191–193]. Single cell RNA-seq studies of human colonic samples identified a subset of IL-11-producing inflammatory fibroblasts, which are also apparent as GFP-labelled IL11 expressing cells in reporter mouse models [192,194–196]. In our own studies, we found that expression of IL11 in stromal cells in mice causes severe colitis and death [92].

## Osteoarthritis

As discussed above, the role of IL11 in bone growth and remodelling is controversial and, in part, derived from studies that used rhIL11 in the mouse and from studies of *Il11ra1* deleted mice [120–123]. Human GWASs have linked a missense variant in IL11, assumed LOF [197], with reduced height and there are further GWAS associations at the locus for osteoarthritis (OA), where the rare variants are linked with greater disease [134,198].

In the earlier literature IL11 levels were found to be elevated in the synovial fluid of patients with OA [199] and IL11 is thought important for bone resorption in cancer metastasis [200]. RNA-seq studies found that *IL11* is 4- and 22-fold up-regulated in subchondral and articular bone in patients with OA [201] and *IL11* up-regulation in OA bone has been replicated in other unbiased genomic screens [202,203].

These collective, genome wide, genetic and transcriptomic human data, replicated across studies, strongly suggest a role for IL11 in OA. The genetic data may suggest LOF as disease-causing and, if true, then the up-regulation of IL11 seen in genomic and proteomic studies would be compensatory. But, this seems at odds with the known disease-causing effects of IL11: fibrosis, inflammation and inhibition of endogenous regeneration. These IL11 toxicities seem even more relevant when one considers that failure of cartilage regeneration is thought to be a key pathology in OA [204]. Furthermore, a gp130 signalling module activated by IL11 is associated with failure of regeneration and OA [149]. Whether IL11 is protective or disease-causing in OA remains to be resolved but there is strong human genetic and genomic evidence for IL11 having an effect in this disease.

## Cancer

The role of IL11 in various forms of cancer, notably gastric, breast, lung and colonic, has been the subject of many reviews and is mentioned briefly here [200,205]. In cancers, IL11 is thought to have both cell autonomous effects in cancer cells and fibroblasts as well as paracrine cross-talk between cells. In the absence of IL11 subclones, the tumour stroma collapses and so does the cancer [206]. Given the strong effects of IL11 on mesenchymal transition in primary cells, it is not surprising that its expression is linked with metastasis [200]. The signalling pathways underlying IL11 effects in cancer were mostly thought JAK/STAT3 related but, based on more recent data, MEK/ERK and — in particular — LKB1/AMPK/mTOR signalling, may now feature. LKB1 is an established tumour suppressor and IL11-mediated inactivation of LKB1, through its sequential phosphorylation at S325A (ERK) and S428A (P90RSK), leads to AMPK inactivation and mTOR activation [73], this sequence of events is linked with cancer cell proliferation [81]. Inhibition of IL11, by a variety of means, is associated with reduced cancer initiation, progression and metastasis and IL11 is being explored as a therapeutic target in combination with immunotherapy for some human cancers by Mabwell Therapeutics (below).

## Rare human genetic diseases

It is interesting to note that IL11 is important for the pathobiology of some rare human genetic diseases where it is up-regulated due to a shared molecular cue or activated because of a generic cellular stress (Table 1).

**Table 1:** IL11 is important for the pathobiology of rare human genetic diseases

Rare genetic disease	Major pathologies	Cells expressing IL11	IL11 effect	Citations
Hermansky Pudlak Syndrome	Lung fibrosis Colitis	Lung ECs and fibroblasts	FMT, EMT, pulmonary fibrosis	[152,154]
Peutz Jeghers Syndrome	Intestinal polyposis Cancer	Intestinal fibroblasts and VSMCs	FMT, intestinal polyposis	[207,208]
Marfan Syndrome	Aortic dissection Lung emphysema	Aortic and lung VSMCs, ECs and fibroblasts	VMT, EndoMT, FMT, aortic dilatation, lung emphysema	[157,178]
Alport Syndrome	Kidney failure Hearing loss	Kidney TECs, podocytes and fibroblasts	EMT, FMT, tubular atrophy, renal fibrosis/failure	[101]

VSMC, vascular smooth muscle cell; TEC, tubular epithelial cell; EC, endothelial cell; EMT, epithelial mesenchymal transition; VMT, VSMC mesenchymal transition; FMT, fibroblast mesenchymal transition; EndoMT, endothelial mesenchymal transition.

In Peutz–Jeghers syndrome, IL11 is up-regulated in colonic stromal cells and linked with colonic polyposis. The reason for IL11 up-regulation in PJS is likely related to heterozygous LOF mutation in LKB1, as LKB1 inactivation is known to stimulate autocrine, mTOR-dependent amplification of IL11 secretion [73,207]. Patients with PJS have an increased risk of cancer and it is interesting to speculate that IL11-mediated activation of wildtype LKB1 [73], on top of the germline, PJS-causing LKB1 LOF mutation, plays a role in cancer risk in these patients. Whether anti-IL11 is effective in treating and/or preventing polyposis and/or cancers in PJS is currently under investigation.

In Marfan Syndrome, IL11 is up-regulated in VSMCs in the aorta and the lung and in this instance IL11 expression is likely due to increased TGFβ activity, which defines Marfan syndrome [157,178]. In a mouse model of Marfan syndrome, anti-IL11 therapy reduced aortic dilation and emphysema of the lungs [157,178].

IL11 is up-regulated in HPS, a disease due to gene mutations that affect biogenesis of lysosome-related organelles complexes leading to lysosomal-related dysfunction that has notable effects in platelets and lung alveolar epithelial cells (AECs) [152,154]. It is thought that gene mutation-driven AEC dysfunction in HPS causes IL11 secretion, autocrine AEC EMT and paracrine activation of fibroblasts, leading to lung fibrosis. HPS patients can also suffer from colitis — perhaps an additional IL11-related pathology.

Alport syndrome is caused by mutation in genes that encode chains of type IV collagen [209]. This causes defects in the glomerular basement membrane causing podocyte dysfunction, glomerular hypertension, and ultrafiltration [210]. IL11 secretion from damaged podocytes, and stressed TECs in particular, causes TEC pEMT and dysfunction, fibro-inflammation, and renal failure [101].

It remains to be seen if IL11 plays a role in the pathogenesis of other rare human diseases, but genetic diseases characterised by increased TGFβ (e.g. Loeys–Dietz syndrome) or mTOR (e.g. tuberous sclerosis) activity may be considered, as may syndromes that activate ROS (e.g. motor neuron disease) or are defined by fibrosis (e.g. cystic fibrosis) or EMT.

## Aging diseases and healthspan

Early on during our studies of IL11 we made the serendipitous observation that Il11 is up-regulated in rodent tissues in old age. This finding stimulated a six-year study (2017–2023) of IL11 in healthspan and lifespan [211]. During this time it became apparent that IL11 is a member of the senescence associated secretory phenotype and that it can directly stimulate senescence in lung fibroblasts and epithelial cells [68,90,212]. IL11 has been linked with photoaging-induced loss of facial subcutaneous fat [213] and SNPs at the IL11 locus are associated with diseases of aging (e.g. osteoarthritis [198] and menopause [214]). IL11 is up-regulated in IPF, common in the elderly [215] and serum IL11 levels are increased in the very old [216]. Our studies of healthspan identified IL11 as an inflammaging factor that causes ERK/mTOR-mediated sarcopenia, metabolic dysfunction and frailty in old mice, while showing that therapeutic targeting of IL11 increases mouse healthspan [211]. Studies of IL11 in lifespan are ongoing and much further research is needed in this new area of IL11 biology.

## IL11 therapeutics

As shown in Figure 1, we have come a long way in our understanding of IL11 and it is now viewed by many as a therapeutic target for fibrotic diseases. This has stimulated the initiation of three phase one clinical trials of anti-IL11 or anti-IL11RA therapy that are in progress (Table 2). These trials all use antibody-based neutralisation of IL11 signalling and it is interesting to see that there are now mice humanised for *IL11* and *IL11RA* (C57BL/6-Il11tm1(IL11)Il11ra1tm1(IL11RA)/Bcgen, Biocytogen), which may be used to screen for therapeutic antibody efficacy in a liver toxicity model, which we first demonstrated [61].

**Table 2:** Clinical trials of anti-IL11 or anti-IL11RA therapy listed on clinicaltrials.gov

Company	Target	Trial ID	Target diseases
Lassen Therapeutics	IL11RA	NCT05331300	Lung fibrosis Thyroid eye disease
Boehringer Ingelheim	IL11	NCT05658107	Lung fibrosis Non-alcoholic steatohepatitis
Mabwell Therapeutics	IL11	NCT05740475	Lung fibrosis Cancer immunotherapy

While safety is yet to be established, data from human and mouse genetics as well as preclinical proof of concept (POC) studies provide hope that inhibition of IL11 signalling will not have serious side effects in adults. The first safety data from NCT05331300 reports an ‘excellent safety profile [sic]’ and this trial has now completed and is moving towards patient dosing [217].

Interestingly, while it is stated that all three phase one trials are forerunners of phase two trials in IPF, Lassen therapeutics is also pursuing thyroid eye disease, which is in part related to IL11-induced FMT [218]. In press releases, Mabwell Therapeutics has mentioned that anti-PD1/anti-IL11 combination therapy appears effective in a variety of solid tumour models, which they plan to test in future trials.

## Conclusion and outstanding matters

This review aimed to summarise the evolution in our understanding of IL11: from the belief it was a haematopoietic and cytoprotective factor to the understanding that it is — in fact — a toxic stimulus. Despite these advances, IL11 remains understudied and there is undoubtedly a lot yet to learn about this little-studied cytokine. An immediate question that will be resolved soon is ‘is anti-IL11/IL11RA therapy safe in humans?’. Equally imminent, is whether rhIL11 should continue to be given to patients with chemotherapy-related thrombocytopenia, as it now has clearly defined and severe cardiac side effects.

In fibro-inflammatory diseases where anti-IL6R therapy effectively targets immune cells but IL11 is also highly up-regulated (e.g. SSc or RA) it is possible that combination therapy with anti-IL11 may improve efficacy, which is an intriguing possibility. Whether targeting IL11 in rare human genetic disease offers an approach for disease mitigation, beyond gene therapy, is unknown but POC in animal models of Alport and Marfan syndrome suggests this premise may be worth testing in clinical trials.

At a more basic level, the detailed mechanism underlying autocrine IL11-induced IL11 protein translation and secretion in stromal cells has yet to be elucidated. The impact of IL11 on the ERK/LKB1/AMPK/mTOR axis needs to be more fully understood, the functional relevance of LKB1 phosphorylation needs to be demonstrated and linked with physiology or pathology. Structural studies of rhIL11 binding to Il11ra1 are needed to further clarify how injection of human IL11 to mice impacts endogenous mouse Il11ra1, which is likely nuanced. The effects of acute, species matched IL11 exposure versus more chronic expression needs greater study. The impact of IL11 in healthspan is intriguing, but findings need to be extended to other age-related pathologies such as cancers, thymic dysfunction, brain disorders and to lifespan. It should be remembered that IL11 is vastly understudied compared with IL6 and there is a lot to learn about this cytokine.

To conclude, IL11 arose in the fish some 400 million years ago to enable a program of epimorphic appendage regeneration that is defined by mesenchymal transitions, cell migration, extracellular matrix production and inflammation. In mammals, this ancient program of regeneration is similarly, but incompletely, activated in response to tissue injury and causes disease rather than repair. Looking forward, it will be exciting to watch the IL11 field as it develops further and to see if the efficacy to anti-IL11 therapeutics seen in murine POC studies translates to patients with either rare or common diseases.

## Abbreviations

AECs: alveolar epithelial cells
AT2: alveolar epithelial type 2
CNTF: ciliary neurotrophic factor
ECs: endothelial cells
EMT: epithelial mesenchymal transition
EndoMT: endothelial mesenchymal transition
FMT: fibroblast mesenchymal transition
GOF: gain-of-function
HPS: Hermansky Pudlak syndrome
HSCs: hepatic stellate cells
IL11: Interleukin 11
IPF: idiopathic pulmonary fibrosis
LIF: leukaemia inhibitory factor
LOF: loss-of-function
OA: osteoarthritis
POC: proof of concept
RA: rheumatoid arthritis
SSc: systemic sclerosis
TECs: tubular epithelial cells
VMT: VSMC mesenchymal transition
VSMCs: vascular smooth muscle cells
